# Supplementary material for: Significant Influence of a Single Atom Change in Auxiliary Acceptor on Photovoltaic Properties of Porphyrin-Based Dye-Sensitized Solar Cells
Source: Nanomaterials (Basel). 2018 Dec 11;8(12):1030. doi: 10.3390/nano8121030 (PMC6316492; doi:10.3390/nano8121030)
Supplement: Supplementary file 1 [file nanomaterials-08-01030-s001.pdf]

Supporting Information

Significant Influence of a Single Atom Change in Auxiliary Acceptor on Photovoltaic Properties of Porphyrin-Based Dye-Sensitized Solar Cells

Haoran Zhou, Jung-Min Ji, Min Su Kim and Hwan Kyu Kim*

Global GET-Future Lab. & Department of Advanced Materials Chemistry, Korea University, 2511 Sejong-ro, Sejong 339-700, Korea; zhouhaoran@naver.com (H.Z.); manbbong@korea.ac.kr (J.-M.J.); kimms38@korea.ac.kr (M.S.K.)

* Correspondence: hkk777@korea.ac.kr

Instrumentation

The ^{1}H NMR and ^{13}C NMR spectra were recorded on a Varian Mercury 300 spectrometer. MALDI-TOF mass spectra were obtained on a Voyager-DETM STR biospectrometry workstation. The UV-visible spectra of porphyrin sensitizers were recorded by a Shimadzu UV-2401PC spectrophotometer. Cyclic voltammetry was recorded by a Versa STAT3 (AMETEK, Germany) instrument. Electrochemical impedance spectroscopy (EIS) measurements were measured with an impedance analyzer (AMETEK). CV experiments were carried out in THF solution with a three-electrode system (an Ag/AgCl reference electrode, a working electrode, and a Pt wire counter electrode) at a scan rate of 50 mV s^{-1} using 0.1M $TBAPF_6$.

DSSC fabrication

The DSSC device fabrication procedure is similar to that of a previous report [1]. The electrolyte was composed of 0.25M $Co(bpy)_3(TFSI)_2$, 0.06M $Co(bpy)_3(TFSI)_3$, 0.1M LiTFSI, and 0.5M 4-tert-butylpyridine in acetonitrile.

Photoelectrochemical measurements of sensitizers and DSSCs

Photovoltaic measurements were performed by a 1000 W Xe light source (Oriel, 91193), which keeps a lamp power of 100 mW/cm^2 at the cell surface. The J_{sc} and V_{oc} were obtained by applying a Keithley model 2400 digital source meter (photocurrent delay time = 40 ms, voltage step = 10 mV).The IPCE spectra were obtained with a75 W xenon lamp (PV Measurements, Inc. IPCE system). A reference Si detector was used to measure the intensity of the monochromatic beam.

Synthetic procedure

Compound 2: To a solution of compound 1 (200 mg, 0.55 mmol) in THF (30 mL) and methanol (15 mL) was added a solution of 20% NaOH (aq) (8 mL). The solution was refluxed for four hours. At this time, TLC (silica, dichloromethane) indicated complete hydrolysis of the ester. The mixture was extracted with dichloromethane (3 × 100 mL), washed with HCl (1 M) and water. After filtration and drying at ambient temperature, the crude dark yellow product **Compound 2** was used to synthesize **SGT-024** without further purification.

SGT-024: To a solution of compound 3 (0.21 g, 0.10 mmol) in THF (15 mL) was added TBAF (1M in THF, 0.31 mL, 0.31 mmol) and the resulting solution was stirred for 30 min at room temperature. Water (30 mL) was added and the organics extracted with CH2Cl2 (3 x 50 mL). The organics were dried (Na2SO4) filtered and evaporated. To the porphyrinic residue was added compound 4 (0.07g, 0.21 mmol, 2 equiv), AsPh3 (0.064 g, 0.21 mmol, 2 equiv.), Pd2(dba)3 (0.019g,

0.02 mmol), THF (15 mL) and Et3N (1.16 mL). The solution was heated at reflux overnight prior to evaporation of the solvents and simple purification by column chromatography (silica, CH2Cl2 / hexane, 2:1) to afford the desired product, **SGT-024**, as a brown solid (0.14g, 63%). 1H-NMR (300 MHz; CDCl3; TMS) δ 9.96-9.94(d, 2H, J = 4.7Hz), 9.41(d, 1H J = 4.7Hz), 9.18-9.16(d, 2H, J=4.7Hz), 8.95-8.94(d, 2H, J = 4.7Hz), 8.89-8.86(m, 2H, J = 8.2Hz), 8.69-8.67(m, 2H, J = 4.7HZ), 8.31-8.28(m, 2H, J = 8.2Hz), 7.74-7.68(d, 2H, J = 9.3Hz), 7.22-7.20(t, 2H, J = 8.8Hz), 3.871-3.809(t, 8H, J = 14Hz), 1.845-1.797(t, 2H, J = 7.3Hz), 1.556-1.531(t, 2H, J = 8.3Hz), 1.48-1.33(m, 8H), 1.32-1.20(m, 16H), 1.01-0.80 (m, 28H), 0.77-0.60 (m, 16H), 0.60-0.38 (m, 16H) 0.55 (t, J = 7.3Hz, 12H). 13C NMR (300 MHz CDCl3): δ 159.701, 156.103, 152.751, 152.365, 151.762, 150.439, 150.015, 146.653, 132.365, 131.765, 131.156, 130.635, 130.274, 129.564, 123.002, 119.823, 114.774, 114.653, 104.841, 77.104, 77.063, 76.952, 76.628, 68.259, 68.087, 31.426, 31.287, 29.264, 28.478, 28.365, 28.287, 25.687, 25.003, 22.487, 22.063, 13.875, 13.698. HR-MS (MALDI-TOF): m/z. calcd for $C_{102}H_{122}N_8O_8SZn$, 1685.5639; found, 1684.7299 (M+).

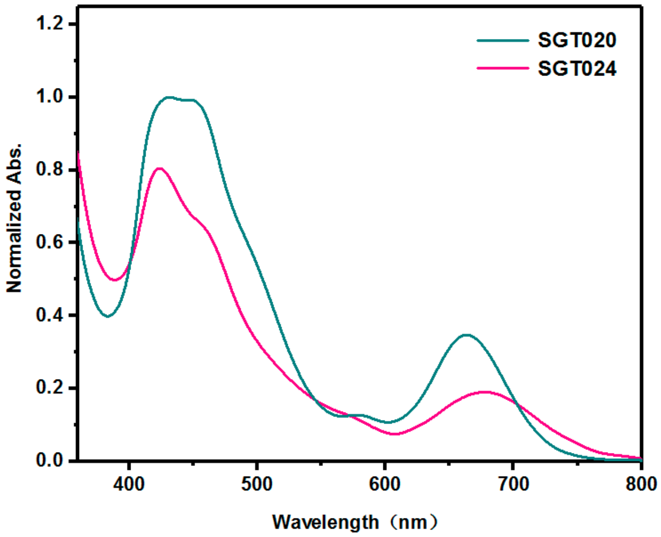

Figure S1. UV spectra of the porphyrin sensitizers on a TiO_2 film (3 μm).

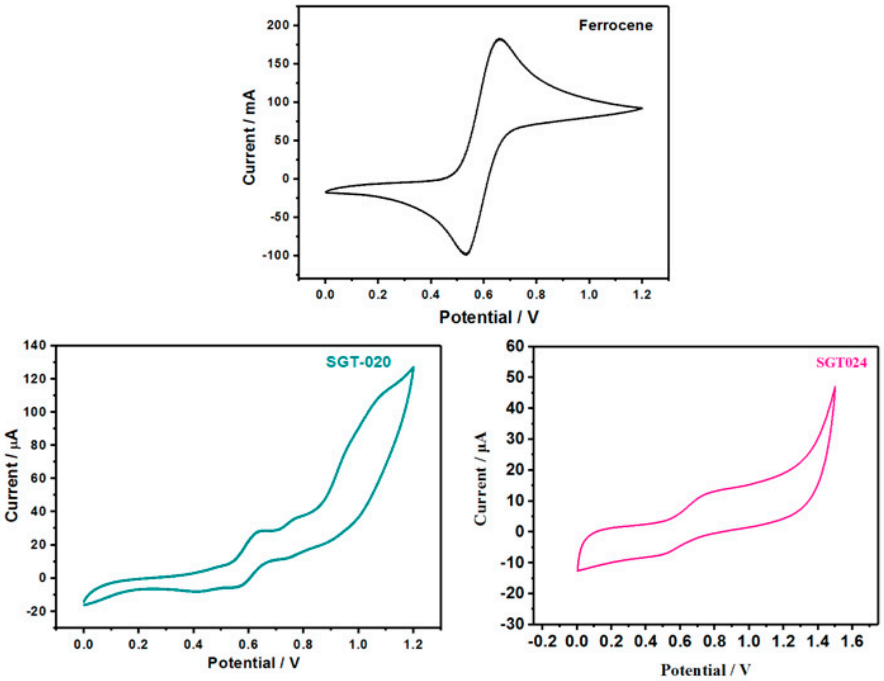

Figure S2. Cyclic voltammograms of **SGT-020** and **SGT-024** in THF/ $TBAPF_6$ and ferrocene external reference.

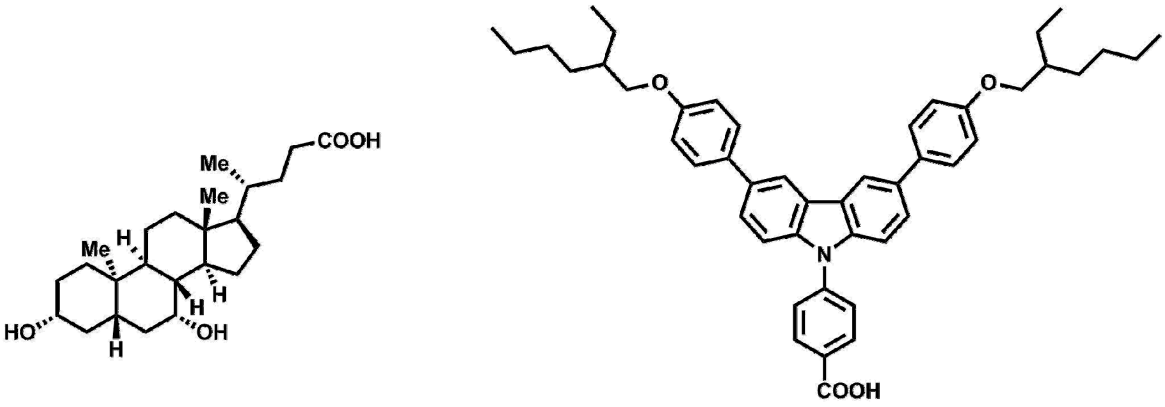

CDCA

HC-A1

Figure S3. Co-adsorbents used in the dye-sensitized solar cells.

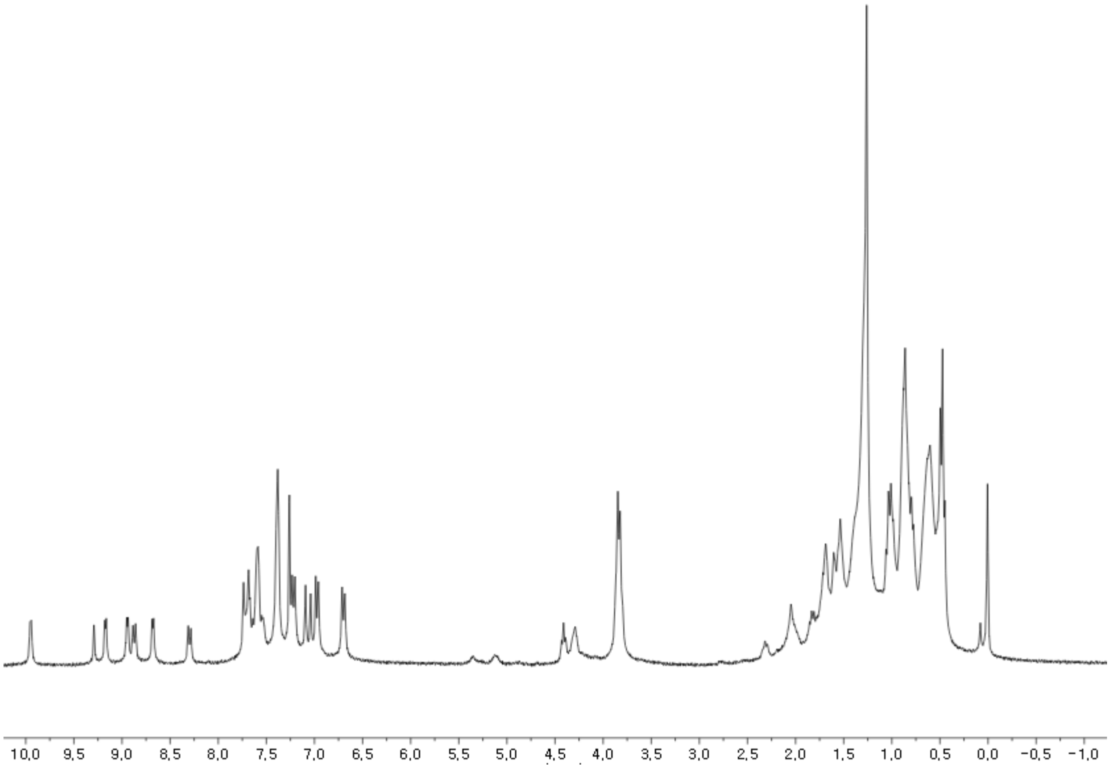

Figure S4. ^{1}H-NMR spectrum (300 MHz, $(CD_3)_2CO$) of **SGT-024**.

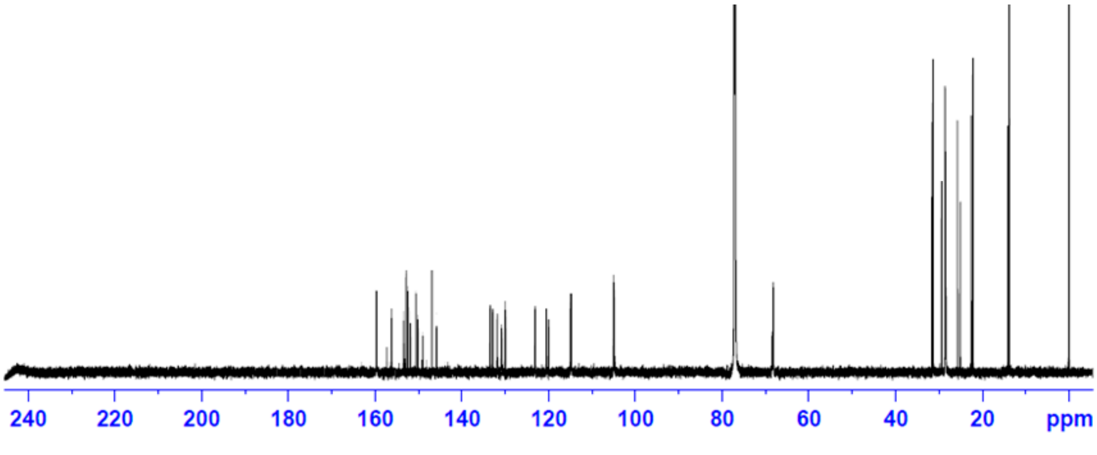

Figure S5. ^{13}C-NMR spectrum (300 MHz, $(CD_3)_2CO$) of **SGT-024**.

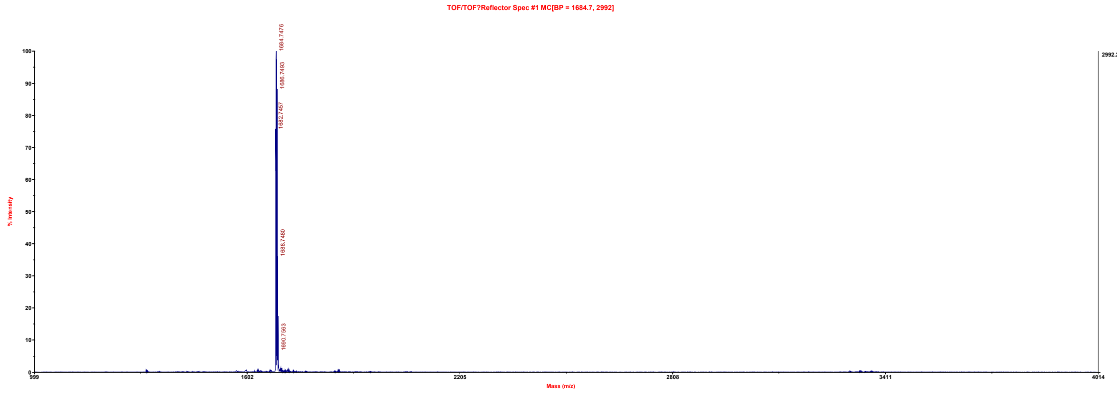

Figure S6. Maldi-TOF spectrum of **SGT-024**.

Table S1. Photophysical and electrochemical properties of porphyrin sensitizers by DFT calculations

	$E_{0\text{-}0}^{\mathrm{DFT}\ a}$ (eV)	$E_{\mathrm{ox}}^{\mathrm{DFT}\ a}$ (eV)	$E_{\mathrm{ox}^*}^{\mathrm{DFT}\ a}$ (eV)	$\lambda_{\mathrm{abs\,max}}^{\mathrm{DFT}\ a}$ (nm)	$f^{\,a}$	MO transition[a]
**SGT-020**	2.22	–5.0	–2.78	427	0.89	H-1→L+1 (32%), H-2→L+2 (22%), H-6→LUMO (16%)
				723	0.69	HOMO→LUMO (88%), HOMO→L+1 (6%), H-2→>L+2 (2%)
**SGT-024**	2.01	–5.02	–3.01	412	0.72	H-5→LUMO (13%), H-2→L+1 (22%), H-2→L+2 (17%), H-1→L+2 (22%)
				781	0.66	HOMO→LUMO (91%), HOMO→L+1 (5%)

[a]Energy gaps ($E_{0\text{-}0}^{\mathrm{DFT}}$), $\lambda_{\mathrm{abs\,max}}^{\mathrm{DFT}\ b}$, *f*, and the corresponding transitions were computed with the TD-DFT method at the M06/6-31G(d) level in THF solvent. Energy gaps ($E_{0\text{-}0}^{\mathrm{DFT}}$) are calculated via $E_{0\text{-}0}^{\mathrm{DFT}} = E_{\mathrm{ox}^*}^{\mathrm{DFT}} - E_{\mathrm{ox}}^{\mathrm{DFT}}$.

Reference

1. Kang, S.H.; Jeong, M.J.; Eom, Y.K.; Choi, I.T.; Kwon, S.M.; Yoo, Y.; Kim, J.; Kwon, J.; Park, J.H.; Kim, H.K. Porphyrin Sensitizers with Donor Structural Engineering for Superior Performance Dye-Sensitized Solar Cells and Tandem Solar Cells for Water Splitting Applications. *Adv. Energy. Mater.* **2017**, *7*, doi:10.1002/aenm.201602117.
